# The role of therapeutic climbing in child and adolescent mental health: a systematic literature review

**DOI:** 10.1186/s13102-025-01417-7

**Published:** 2025-11-24

**Authors:** Carmen Kaar, Anika Frühauf, Martin Kopp

**Affiliations:** Department of Sport Science, University of Innsbruck, Fürstenweg 176, Innsbruck, 6020 Austria

**Keywords:** Climbing, Bouldering, Mental health, Psychological well-being, Children and adolescents, Youth, Physical activity intervention, Exercise therapy

## Abstract

**Background:**

Therapeutic climbing is increasingly applied as a physical activity intervention targeting mental health in children and adolescents. However, the evidence base regarding its effectiveness and implementation remains fragmented. This systematic review aimed to synthesize current evidence on the effects of therapeutic climbing on mental health and interrelated psychological domains in children and adolescents.

**Methods:**

The review followed PRISMA guidelines and was registered in PROSPERO. Seven electronic databases (Pubmed, PsycINFO, PsycARTICLES, PubPsych, Medline, Web of Science, and CINAHL) were systematically searched for controlled trials investigating therapeutic climbing in children and adolescents, focusing on outcomes on mental health and interrelated domains such as affective and emotional responses, self-efficacy and social dimensions. Study selection, data extraction, and risk of bias assessment were conducted independently by two reviewers.

**Results:**

Five controlled trials met the inclusion criteria, including three randomized controlled trials and two non-randomized studies. Significant heterogeneity in study designs, intervention characteristics, and outcome measures limited comparability across studies. Reported benefits included improvements in overall mental health, psychological distress, self-efficacy, alienation, and affective and emotional states. However, reliance on wait-list controls, the absence of active control groups, small sample sizes, and potential expectancy biases complicated the interpretation of findings. Additionally, variability in group sizes, facilitator roles, and intervention settings highlighted the need for standardized protocols to enhance consistency and reliability in future research.

**Conclusions:**

Therapeutic climbing showed promise as a psychologically beneficial intervention for children and adolescents. However, the current evidence base is limited, with few studies and significant variability in study designs, populations, and outcome measures. Notably, it remains unclear whether therapeutic climbing is superior to other physical activity interventions. Future research should address methodological limitations, incorporate active control groups to disentangle climbing-specific effects from general physical and social benefits, and investigate the mechanisms underlying observed effects. Greater emphasis on clinical populations and diverse settings will enhance the evidence base and guide practical applications.

**Trial registration:**

The systematic review was registered in the International Prospective Register of Systematic Reviews (PROSPERO) (registration number: CRD42024619108)

**Supplementary Information:**

The online version contains supplementary material available at 10.1186/s13102-025-01417-7.

## Introduction

Physical activity (PA) is associated with several health benefits, including improved cardiovascular health [1], enhanced musculoskeletal strength [2, 3], and better metabolic function [4], both in children and adults. It also serves as a protective factor against somatic illnesses and pathological behaviours, reducing the risk of chronic conditions such as type 2 diabetes [5], hypertension [6], or obesity [7], while supporting overall long-term health [8–10], including significant positive effects on mental health [11, 12]. Evidence suggests that children and adolescents who regularly engage in PA demonstrate increased self-esteem [13, 14], higher cognitive functioning, more positive affect and mood [15], and experience feelings of competence, confidence, and social interaction and relatedness [16]. Further, PA also serves as a protective factor against mental health disorders such as depression or anxiety [17]. In addition to its preventive benefits, PA is used as an accessible mental health intervention that is cost-effective, has minimal or even beneficial physiological side effects, and, in certain cases, demonstrates effectiveness comparable to pharmacological treatments [18].

Childhood and adolescence are formative periods for the development of various psychological domains, in addition to being times of increased vulnerability to mental health problems. Epidemiological data show that about half of all lifetime mental health disorders emerge by mid-adolescence, highlighting the importance of implementing early preventive and therapeutic interventions during these stages [19]. Ongoing neurodevelopment, particularly the prolonged maturation of executive control systems [20, 21] and the dynamic reorganization of social-affective brain networks [22], shapes how young people acquire self-regulation skills, build self-efficacy, and form social connections [22].

In recent years, climbing as a recreational activity became extremely popular, both among youth and adults [23]. Climbing is a unique form of resistance training combining physical, psychological, and social demands in an exceptional and engaging manner. Climbing requires strength, flexibility [24], endurance [25], postural stability [26, 27], balance and coordination [27] while fostering psychological skills such as problem-solving, resilience, and social cooperation [28]. It also requires sustained attention and cognitive control (route planning), provides repeated mastery experiences that build self-efficacy [28], and is frequently practiced in cooperative, social settings that could foster interpersonal connectedness. Unlike traditional team sports, climbing minimizes competition and emphasizes individual achievement, which can enhance self-esteem and create a sense of accomplishment [28]. Due to its multidimensional demands, climbing has gained significant attention as a therapeutic approach in clinical and non-clinical settings [29, 30]. Therapeutic climbing, often conducted on artificial climbing walls, involves activities such as bouldering and top-rope climbing that are adaptable to individual skill levels and needs. Apart from its use to improve and maintain physical fitness such as gait function in children with cerebral palsy [24], climbing therapy is also applied to improve mental and social health [30, 31]. Climbing is increasingly being incorporated into interventions for improving mental health outcomes, as it promotes several domains interrelated with mental health such as executive functions, enhanced focus, perceived self-efficacy, or social connectedness - each associated with better mental health outcomes in youth [27, 28, 32, 33]. However, despite its growing use, evidence supporting its effectiveness remains unclear, particularly among children and adolescents. Especially the effectiveness of climbing therapy compared to other PA interventions aiming at improving mental health, should be discussed.

Much of the existing research on therapeutic climbing has focused on adult populations, with limited studies specifically addressing its impact on younger age groups. A recent systematic review [34] has synthesized the evidence on therapeutic climbing, concluding that climbing may improve physical, mental and social outcomes but highlighted important evidence gaps and heterogeneity in populations and methods [34]. The developmental characteristics of childhood and adolescence suggest that children and adolescents may respond differently to psychological and behavioural interventions than adults, making it inaccurate to assume that findings from adult populations will directly translate to younger age groups. Given this, a focused synthesis of the evidence for mental health and interrelated psychological domains in children and adolescents is warranted - both to clarify current effects and to identify priorities for future, developmentally informed research.

The aim of this systematic literature review was to explore and evaluate the existing evidence on the relationship between climbing interventions and the mental health and interrelated psychological domains of children and adolescents. In addition, this review aimed to explore how therapeutic climbing interventions compare to other physical activity-based interventions and psychosocial or therapeutic approaches. By synthesizing current research, this review sought to identify gaps in knowledge, provide recommendations for future research, and support the development of evidence-based practices for using climbing as a therapeutic intervention in children and adolescents.

## Method

The systematic review was conducted and reported according to the PRISMA (Preferred Reporting Items for Systematic Reviews and Meta-Analyses) guidelines [35]. The systematic review was registered prospectively in the International Database for Prospective Register of Systematic Reviews (PROSPERO, www.crd.york.ac.uk/PROSPERO/) on December 2nd 2024 (registration number: CRD42024619108) and a full study protocol was developed prior to conducting the review, which is available upon request. Only peer-reviewed studies published in English or German language were included. All tasks were carried out by two independent researchers. In case of disagreement, discussion and consensus were followed, or a third researcher (M.K.) was involved in resolving the discrepancies.

### Information sources and search strategy

A literature search was conducted on 3rd of December 2024 in the electronic databases Pubmed, PsycINFO, PsycARTICLES, PubPsych, Medline, Web of Science, and CINAHL. The search term consisted of keywords identified through preliminary searches and reviews on similar topics. The final search strategy was peer-reviewed by a co-researcher (M.K.). The search comprised of three elements: population (children, adolescents), intervention (climbing), and outcome (mental health, psychological well-being, etc.). For an outline of the search strategy, please see supplementary material. Furthermore, manual forward and backward searches through the reference sections of the included articles and relevant reviews were conducted to identify additional articles that were not found during the initial database search. This search was conducted without imposing any restrictions on the date range. An example of the search string can be found in the supplementary materials.

### Eligibility criteria

Eligibility criteria were developed according to the PICOS scheme (i.e., Population, Intervention, Comparison, Outcome, Study design) [36].

#### Population

Studies that examined participants aged 0–18 years were included in the review, consistent with the United Nations Convention on the Rights of the Child´s definition of children [37]. The population was not restricted to individuals with any specific physiological or psychological conditions. Both, typically developing children and adolescents and those with special needs such as physiological or psychological impairments were included. Studies focusing exclusively on adults (>18 years) were excluded.

#### Intervention

Climbing, including bouldering, sport climbing, rock climbing, had to be the main intervention in included studies. Studies were considered as eligible if they contained climbing either as a therapeutic or educational intervention. Short- and long-term climbing interventions were considered as relevant as recent research emphasizes the effects of acute bursts of PA on psychological parameters and mental health [38, 39]. Therefore, climbing interventions consisting of only a single session were also deemed eligible for inclusion in this review. Interventions in which climbing was not the primary activity were excluded.

#### Comparison

Only studies utilizing a control group or control condition were included in this review. Eligible control conditions included: (1) no intervention or treatment, where participants in the control group did not receive any specific intervention, including wait-list control groups; (2) alternative PA, where participants engaged in a form of PA other than climbing; and (3) standard treatment, such as therapy or treatment as usual commonly applied in clinical or practical settings. These criteria were established to ensure that the effects of climbing interventions could be appropriately compared against relevant control conditions. Single-group designs without a control condition were excluded to ensure comparability of outcomes.

#### Outcome

The selection of outcome domains was based on pre-defined conceptualizations of mental health in children and adolescents, drawing on frameworks such as that proposed by Lubans et al. [32]. This framework emphasizes the interrelated nature of cognitive function, psychological well-being, and psychological ill-being (i.e., internalizing and externalizing problems) as important determinants of overall mental health in youth [32]. Accordingly, studies were included if they evaluated outcomes related to any of these domains. This encompassed a broad range of constructs, including cognitive functioning (e.g., executive function, attention, working memory), symptoms of anxiety, depression, stress, self-esteem, self-efficacy, resilience, emotional regulation, social dimensions and overall quality of life. Studies assessing exclusively physical or physiological outcomes (e.g., strength, endurance, or motor performance) were excluded.

#### Study design

Included study designs comprised experimental and quasi-experimental designs, such as randomized controlled trials (RCTs), non-randomized controlled trials (NRCTs), and cross-over designs. Non-controlled trials, such as prospective single-group studies and case studies/case series, systematic reviews, scoping reviews, meta-analysis, qualitative studies as well as dissertations and unpublished literature were excluded.

### Study selection

All articles identified through the database search, including their titles, abstracts, and full texts where available, were uploaded into the EPPI Reviewer [40]. Duplicates were subsequently removed. Titles and abstracts were screened independently by two reviewers to assess eligibility based on predefined inclusion criteria. Full texts of potentially relevant articles were then retrieved and evaluated. Disagreements at any stage were resolved through discussion.

### Risk of bias in included studies

The quality, internal validity, and risk of bias of the studies were evaluated using established tools. For RCTs, the Cochrane Collaboration’s Risk of Bias Tool (RoB-2) [41] was utilized, which assesses five domains: randomization process, deviations from intended interventions, missing outcome data, outcome measurement, and selection of reported results. Each domain was rated as “low risk,” “high risk,” or “some concerns” by two independent researchers, with disagreements resolved through discussion. For the two NRCTs, the Risk of Bias In Non-Randomized Studies—of Interventions (ROBINS-I V2) tool [42] was employed. This tool evaluates seven domains: confounding, classification of interventions, selection into the study, deviations from intended interventions, missing data, measurement of outcomes, and selection of reported results. Similar to RoB-2, each domain was rated as “low,” “moderate,” or “high” risk by two researchers, and an overall risk of bias was assigned to each study.

### Data extraction

For data extraction, the Data Collection Form for Intervention Reviews – Randomized and Non-Randomized Trials from the Cochrane Collaboration [43] was utilized. Data from the included studies were systematically extracted and organized into study characteristics. A single reviewer performed the extraction, which was then verified by a second reviewer for accuracy. Extracted data included information on study design, population characteristics, recruitment, intervention characteristics, control conditions, outcome measures and assessment periods. To structure the diverse outcomes reported across studies, results were categorized according to the framework by Lubans et al. (2016), which conceptualizes mental health as an overarching construct encompassing multiple interrelated domains. These included: (1) psychological ill-being (e.g., emotional distress, internalizing and externalizing symptoms), (2) affective and emotional outcomes (e.g., mood states, exercise-induced mood changes), and (3) additional domains such as self-perception, self-control, self-efficacy, and social dimensions. While interrelated, this categorization enabled a clear and conceptually consistent synthesis of the evidence. A meta-analysis was not conducted in this review due to substantial heterogeneity among the included studies, particularly in terms of study designs, intervention durations, and outcome measures [44].

## Results

### Results of the search

**Fig. 1:**
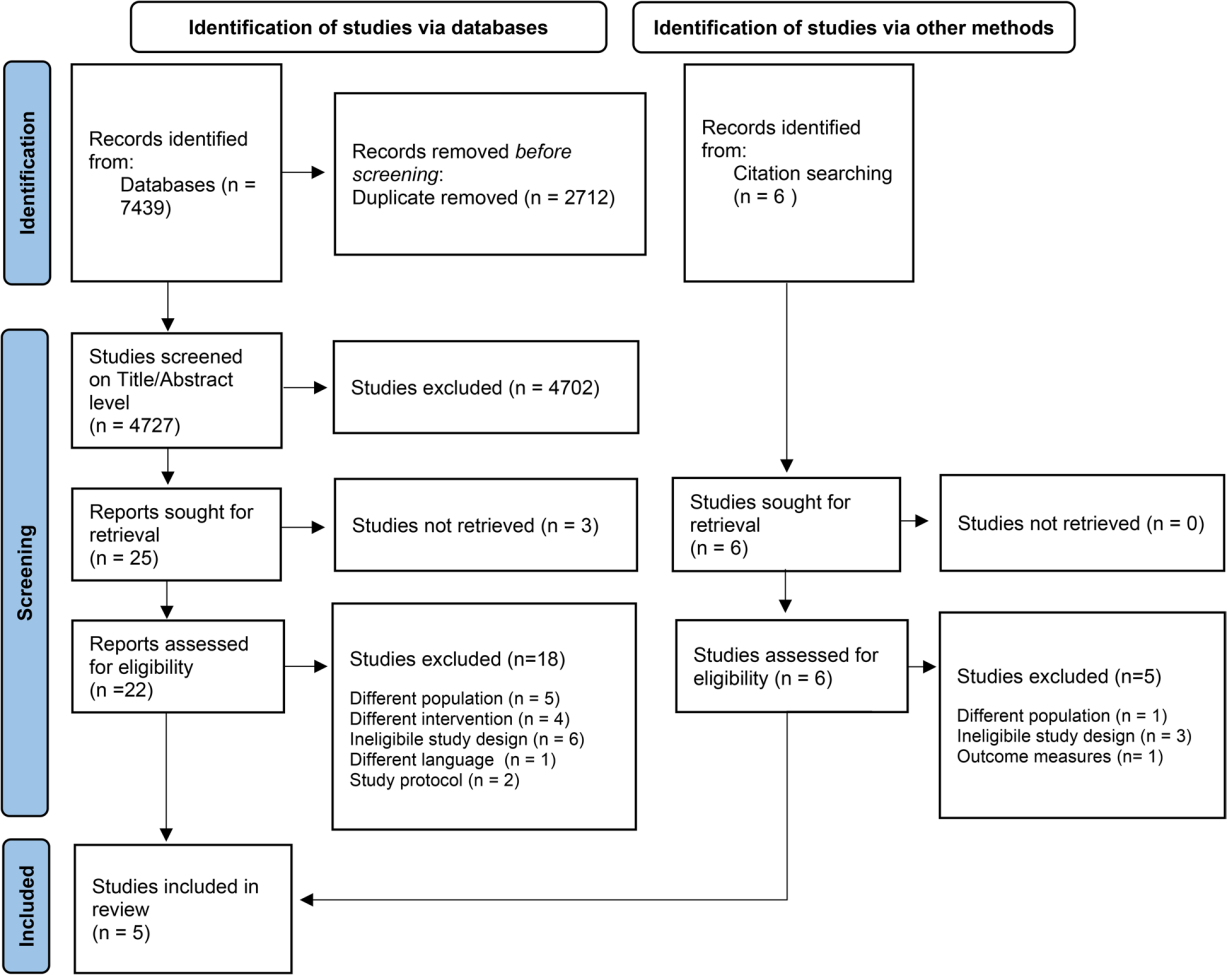
Flow Chart of literature search and study selection process

The initial database search yielded 7439 records, which were reduced to 4727 after duplicate removal. Following title and abstract screening, 22 articles were selected for full-text review. Six additional articles were identified through reference searches. Ultimately, five studies met the inclusion criteria [39, 45–48]. A detailed overview of this selection process is presented in Fig. 1. An overview of the characteristics of the included studies is provided in Table 1.

**Table 1:** Study characteristics

**Author**	**Year**	**Study design**	**N**	**Population**	**Age mean ± SD (range)**	**Recruitment setting**	**Intervention**	**Duration**	**Group Size **	**Facilitators per Group**	**Control condition**	**Outcome measures (Instruments)**	**Measure points**
Cross, R.	2002	NRCT	34	at-risk adolescents	n.r. [12–19]	alternative high school	rock-climbing camp	5 days	4	1	no intervention	Perception of Alienation (Dean Alienation Scale);Perception of personal control (The New Multidimensional Measure of Children´s Perceptions of Control)	pre-/post-intervention
Frühauf et al.	2020	cross-over	33	inpatient psychiatric children and adolescents	13.3 ± 2.2 [10–18]	inpatient psychiatric unit	rope climbing, bouldering	single session (60min)	2–5	2	swimming, occupational therapy	Affective Valence (Feeling Scale); Perceived Activation (Felt Arousal Scale); Positive and Negative Affect (PANAS-C); Subjective Exercise Intensity (RPE)	Affective Valence and Activation: pre-, 20/40 min, post-intervention; Positive and negative Affect: pre-/post-intervention; RPE: every 20 minutes
Krüger & Seng	2019	RCT	78	8th grade students	14.41 ± 0.71 (n.r.)	secondary school	rope climbing	2x3 hours in 1 week	2	-	wait-list control	Barriers Self-Efficacy in Climbing (specifically designed questionnaire); Barriers Self-Efficacy in Belaying (specifically designed questionnaire)	pre-/post-intervention
Luttenberger et al.	2024	quasi RCT	233	adolescents in Bekaa Valley in Lebanon	16.05 ± 1.57 [14–19]	via multiple channels (schools, NGOs, social media,…)	bouldering	2 hours/week for 8 weeks	up to 12	5–6	wait-list control	Mental Well-Being (WEMWBS);Psychological Distress (K-6); Self-Efficacy (GSE); Social Cohesion (modified ARK survey)	pre-/post-intervention
Mazzoni et al.	2009	RCT	46	children with special needs	8.4 ± 1.7 [6–12]	outpatient therapy	rope climbing	1 hour/week for 6 weeks	1–2	1	wait-list control	Self-Efficacy (specifically designed questionnaire); Efficacy rated by belayers (specifically designed checklist); Climbing height, route difficulty (rated by belayer); Self-Perception of athletic & social competence, self-worth (SPPC)	Self-Efficacy: 1 st and 6th session; Belayer´s efficacy ratings: 1st, 3rd and 6th session; Climbing height and difficulty: every session; Self-perception: pre-/post-intervention

This table shows the study characteristics of the 5 included studies

*Abbreviations:* *NRCT* Non-randomized controlled trial, *RCT* Randomized controlled trial, *n.r.* Not reported

### Study design

All included studies were published between 2002 and 2024 in five different journals and were reported in English language. Two studies [45, 48] were conducted in the United States, one study in Austria [39], one study in Germany [46] and one in Lebanon [47]. Three studies were RCTs [46–48], whereas it must be considered that within one study [47] only 65% of the participants were randomly assigned due to cultural and logistical constraints in some cases. One study was a NRCT [45], in which conventional matched-pair design was applied, and one study was designed as a non-randomized cross-over study [39].

### Participant characteristics

Sample sizes ranged from 33 [39] to 233 [47] participants and all studies recruited both sexes, with ages spanning 6–19 years. Three studies focused on adolescents only. Two studies recruited students, from which one focused on at-risk adolescents [45] and the other study included 8th grade adolescents without any special needs [46]. Luttenberger et al. [47] studied young refugees and local adolescents in Lebanon with average mental health but increased psychological distress at baseline. Two studies involved clinical populations: Frühauf et al. [39] examined in-patient psychiatric care patients with various mental health disorders (e.g., depression, emotional disorders, reactions to stress, adjustment and conduct disorders, ADHD, personality disorders, eating disorder), and Mazzoni et al. [48] studied children with special needs undergoing physiotherapy or occupational therapy due to delayed motor development.

### Intervention characteristics

All trials used some type of climbing as the primary intervention. For example, Luttenberger et al. [47] applied bouldering, while Frühauf et al. [39] combined bouldering and rope climbing. The other three trials applied rope climbing [45, 46, 48]. Climbing took place in various contexts, including schools [45, 46], inpatient psychiatric care [39], outpatient programs [48], and community interventions [47]. Intervention structures varied: Krüger and Seng [46] provided unstructured student-led rope climbing, while the study by Cross [45] integrated climbing with psychosocial processing in a structured five-day camp in the nature. The five-day camp combined top-rope climbing, stretching, and runs with guided group discussions, individual journal writing, and technical instruction (e.g., knots, anchors, rappelling). Frühauf et al. [39] implemented a structured climbing program in an indoor gym, combining rope climbing and bouldering with individualized support. The YouCLIMB intervention by Luttenberger et al. [47] featured a structured bouldering therapy program emphasizing resilience and interpersonal skills through experiential activities. Mazzoni et al. [48] tailored a highly individualized approach, incorporating safety training and social skill development. Intervention durations varied from a single 60-minute session [39] to an eight-week program [47]. For details on session length and frequency, see Table 1.

### Facilitators and training

Facilitators included schoolteachers [45, 46], therapists [39, 48], and a multidisciplinary team in the ClimbAID intervention [47]. Cross [45] specified that the teachers had previously completed a technical rock climbing class and were outdoor education majors, while Krüger and Seng [46] involved a physical education teacher climbing gym staff support. Frühauf et al. [39] emphasized that the therapists completed previously a training for climbing therapy in mental health institutions. ClimbAID facilitators were trained in psychosocial support, child protection, and climbing-specific instructions [47].

Group sizes varied across studies, ranging from small, individualized sessions [48] to larger groups in school-based interventions [46]. Similarly, facilitator-to-climber ratios differed, with some studies employing a high level of supervision, such as one facilitator per participant [39] while others used a lower ratio in more autonomous climbing settings [46]. For further details, please see Table 1.

### Control conditions

In the included studies, control conditions varied across research. Three studies used non-treatment control groups (CGs) [45–47], with the latter two employing wait-list controls. Mazzoni et al. [48] also used a wait-list design but did not specify concurrent therapies. In the rock-climbing study, the CG consisted of students who chose not to participate in the climbing program but volunteered to take part in the study in another capacity [45]. Frühauf et al. [39] applied a cross-over design, comparing climbing with swimming and occupational therapy. All interventions were equivalent in duration, but climbing and swimming were conducted in group settings, while occupational therapy was provided as an individualized 1:1 intervention. In general, it must be considered that participants in the study by Frühauf et al. [39] likely received other treatments concurrently as interruptions on conventional treatment programs were kept at a minimum. Yet, it was not reported which other therapies and interventions the participants received parallel [39].

### Risk of bias

Risk of bias assessment of included RCT is displayed in Table 2. One RCT [46] was rated as low risk, while the other two [47, 48] had some concerns. In the study by Mazzoni et al. [48], concerns were raised due to potential differences in treatment between the intervention and control groups, as well as the administration of a self-perception instrument to only a subset of participants. In Luttenberger et al. [47], partial randomization due to logistical and cultural constraints led to some concerns.

**Table 2:**
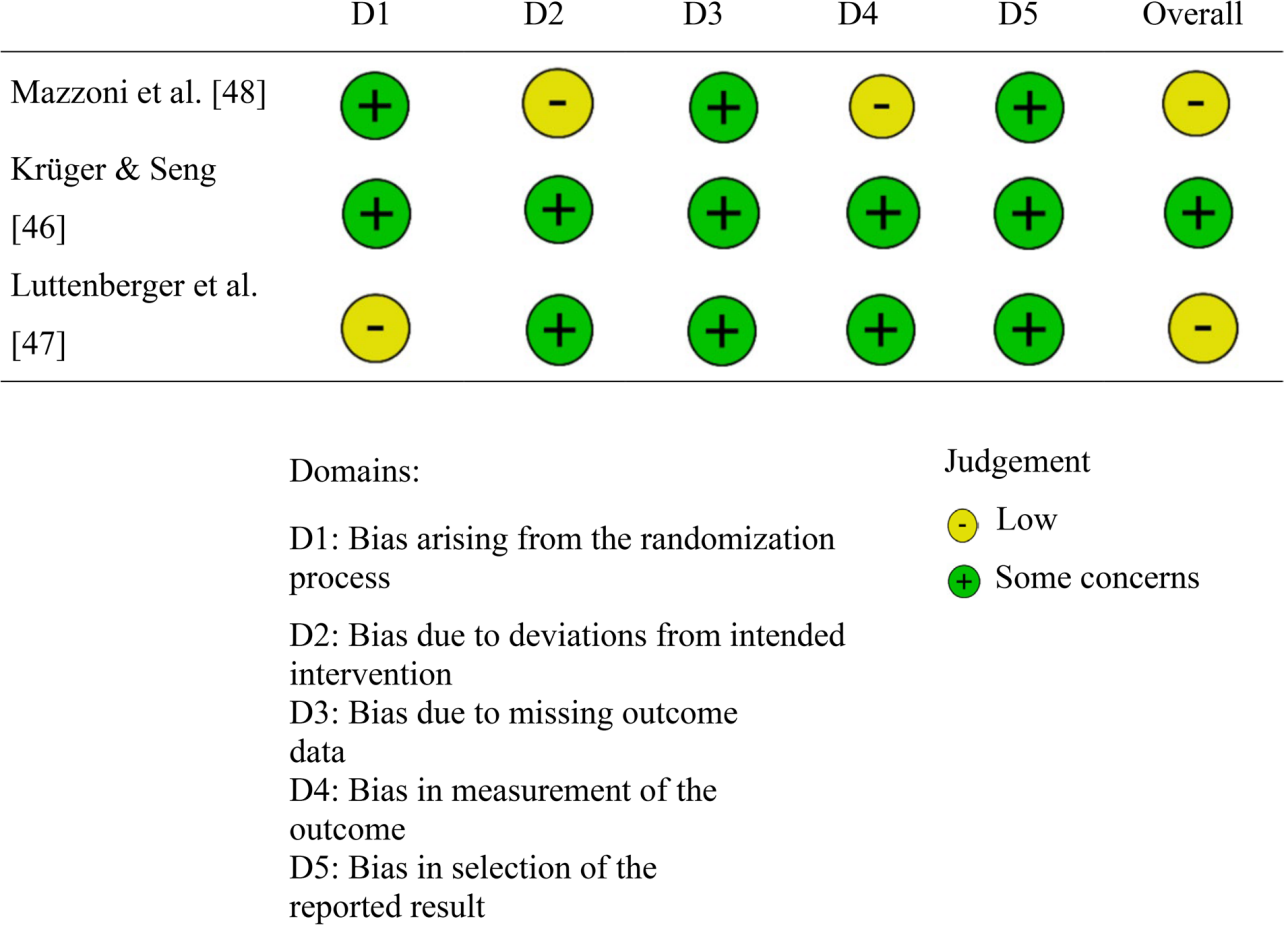
Risk of bias assessment of rCT [46–49]

The two non-randomized controlled trials (see Table 3) were both rated as having a moderate risk of bias. In the study by Cross [45] several factors contributed to this rating. The “bias due to confounding” was judged as moderate risk because, although the study matched intervention and control groups on factors like age and gender, the lack of randomization and potential unidentified confounders posed a risk. The “bias in selection of participants” was also rated as moderate risk due to the volunteer-based selection from an alternative high school and the potential selection bias from matching without randomization. Additionally, the “missing outcome data” was classified as moderate risk due to a drop in intervention group participants, and the “bias in measurement of outcomes” was deemed moderate risk due to low reliability in some subscales of an applied scale.

**Table 3:**
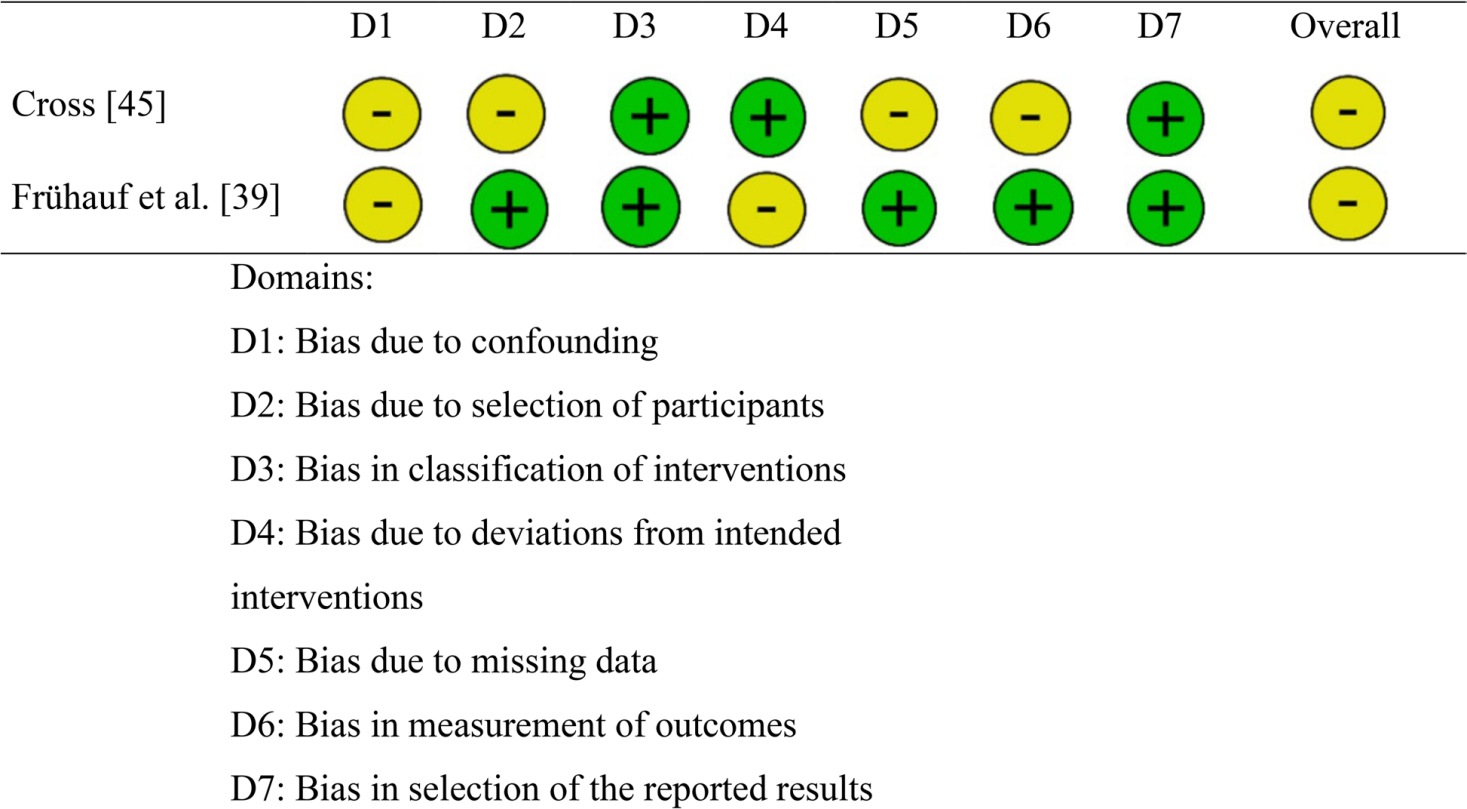
Risk of bias assessment of NRCT [39, 45, 49]

Similarly, in the study by Frühauf et al. [39], the “bias due to confounding” was rated as moderate risk because the study did not account for the potential impact of differing mental health diagnoses on responses. The “deviations from intended interventions” were also classified as moderate risk, as one control intervention (swimming) was conducted in a public pool, potentially introducing uncontrolled environmental factors.

### Effects of interventions

#### Overall mental health

Luttenberger et al. [47] measured emotional and functional aspects of mental health as primary outcome using the Warwick-Edinburgh Mental Well-Being Scale (WEMWBS) [50]. Significant improvements in overall mental health in the intervention group (IG) compared to the control group (CG) were revealed [47].

#### Psychological ill-being

Luttenberger et al. [47] assessed psychological ill-being, specifically the main depressive symptoms (e.g., nervousness, despair, feelings of worthlessness)by using the Kessler psychological distress scale (K-6) [51]. Reductions in psychological distress in the IG compared to the CG were found [47].

#### Self-perception and self-control

Two studies assessed outcomes related to self-perception and self-control, including locus of control and perceived competence. In the study by Cross [45] locus of control was evaluated through Connell’s New Multidimensional Measure of Children’s Perceptions of Control [52], focusing on internal control, powerful others’ control, and unknown control across general, physical, social, and cognitive domains. The results showed a significant increase in personal control in the experimental group after the intervention, compared to their pre-intervention scores,,with no significant differences across gender, ethnicity, or family composition [45]. Mazzoni et al. [48] used Harter´s Self-Perception Profile for Children [53] measuring the three subscales of athletic competence, social competence, and global self-worth. No significant group differences were found in these three domains [48].

#### Affective and emotional responses

Frühauf et al. [39] explored acute affective and emotional responses to therapeutic climbing in children and adolescents. Affective valence was measured using the Feeling Scale [54], and perceived activation was assessed with the Felt Arousal Scale [55]. Positive and negative affect were evaluated through the Positive and Negative Affect Scale-Children [56]. Ratings of Perceived Exertion [57] assessed subjective exercise intensity. The climbing intervention led to a significant increase in positive affect and a decrease in negative affect from pre- to post-intervention. Affective valence and perceived activation also showed consistent increases throughout the climbing session, indicating enhanced emotional well-being and engagement [39].

When compared to other interventions such as swimming and occupational therapy, climbing demonstrated a stronger increase in affective valence during the initial 20 min compared to swimming, and during the first 40 min compared to occupational therapy. Additionally, participants in the climbing group reported significantly higher perceived activation and ratings of perceived exertion than those in the comparison groups [39].

#### Self-Efficacy

Two studies assessed self-efficacy as the main outcome, both using specifically developed climbing-specific questionnaires based on Bandura´s social cognitive theory (SCT) [58]. Although task-specific self-efficacy may seem narrowly focused, Bandura’s SCT [58] posits that self-efficacy beliefs influence motivation, resilience, and emotional regulation, all key components of mental health [32]. Evidence suggests that gains in task-related self-efficacy can transfer to improved general self-confidence and coping abilities beyond the specific activity [59, 60]. Both studies explored self-efficacy related to climbing and belaying, yet, Krüger and Seng [46] used only adolescents´ self-ratings while Mazzoni et al. [48] included a questionnaire self-rated by the participants and a checklist answered by belayers. Krüger and Seng [46] focused specifically on barriers self-efficacy, which refers to the perceived ability to perform a task despite facing obstacles. In the context of climbing, it relates to an individual’s confidence in their ability to keep climbing despite challenges like a fear of heights, physical exhaustion, or tough climbing conditions. For belaying, barriers self-efficacy refers to the perceived ability to manage and support a climber safely, despite concerns such as emotional stress, responsibility for the climber’s safety, or the physical demands of belaying [61]. In this study, two newly developed self-efficacy scales were used to assess barriers self-efficacy for climbing and belaying. For the developed scales measuring barriers self-efficacy of climbing and belaying, a higher score indicated a higher level of barriers self-efficacy, with ratings ranging from 0% (not sure at all) to 100% (most sure) [46]. For barriers self-efficacy of climbing, although no significant group by time effect was found, the IG displayed a slight positive increase in barriers self-efficacy over time, whereas the CG remained stable. For belaying, a significant group by time interaction was found, with the IG demonstrating a substantial increase in barriers self-efficacy of belaying, while the CG showed a minor decrease. In the study by Mazzoni et al. [48] children reported a significant increase in self-efficacy toward climbing from pretest to posttest. This finding was in line with belayers ratings, showing also significant improvements in children´s efficacy over time. Luttenberger et al. [47] also investigated global self-efficacy as secondary outcome and participation in the bouldering program increased self-efficacy, but this finding was not significant.

#### Social dimensions

Social connectedness and cohesion are key determinants of mental health in children and adolescents [62–64]. Two studies in this review assessed outcomes related to social aspects, including alienation [45] and social cohesion [47]. Cross [45] measured alienation using the Dean Alienation Scale (DAS) [65], which assesses powerlessness, normlessness, and social isolation. The IG showed a significant reduction in alienation compared to their pre-intervention scores [45]. Luttenberger et al. [47] assessed social cohesion using two subscales from the modified Attitude, Reactions, and Knowledge (ARK) Regular Perception Survey [66]. One subscale measured scenarios of contact between members of different nationalities (e.g., school, workplace, living in proximity) and the second subscale assessed shared values and lifestyles between Lebanese and Syrians. In this specific case where two ethnic groups (Lebanese and Syrian refugees) were brought together, social cohesion showed neither significant improvements nor a decline through the intervention [47].

## Discussion

This systematic review investigated the impact of climbing interventions on mental health and psychological outcomes in children and adolescents. While the findings of five included papers suggest that climbing can positively influence mental health and interrelated psychological domains, such as self-efficacy, and emotional regulation, the evidence is still limited and requires further exploration.

The present systematic review exclusively included controlled trials - both randomized and non-randomized - that met pre-set inclusion criteria, focusing on children and adolescents and outcome measures on mental health. Only five studies qualified for inclusion, reflecting the limited availability of high-quality evidence on the psychological effects of (therapeutic) climbing in youth populations. It is worth noting, however, that there is a broader body of literature comprising uncontrolled trials [67–69], case studies [70], and publications sharing practical experiences and qualitative insights [71, 72]. These reports provide valuable insights into the application of therapeutic climbing for various psychological and behavioral disorders, such as improving attention, behavioral regulation, and executive functioning in children with ADHD [67], or enhancing social skills in children with developmental delays [68]. Participants with mental disorders reported improvements in psychological well-being, mood, coping, self-reflection, and social connection [73]. These mental health benefits often transfer to daily life, aiding social functioning and school performance [72]. Although some studies lack large-scale rigor, their findings provide valuable insights into the mechanisms and therapeutic potential of climbing, complementing controlled research and contributing to a holistic understanding of its impact on youth mental health.

The five studies in this review varied significantly in design, measures, and interventions, showing climbing’s adaptability across populations, from those with special needs to high school students. This aligns with the findings of Gassner et al. [34] on climbing’s versatility in improving physical, mental, and social health, mainly in adults. While physical benefits like enhanced fitness and balance are well-documented, mental and social effects, including reduced anxiety and improved self-efficacy, are less consistent. These inconsistencies arise from differences in study designs, intervention lengths, and outcome measures, making it challenging to pinpoint the most effective intervention strategies.

Interest in the psychological benefits of climbing has been increasing as demonstrated in a recent review by Mangan et al. [74]. The review emphasizes that climbing enhances intrinsic motivation and promotes flow states in both experienced and novice climbers. Additionally, it highlights climbing’s positive effects on self-confidence, decision-making, and risk management - key factors in clinical interventions targeting mental health improvements. Although climbing interventions are frequently applied in psychiatric settings [31, 75], only one included study in the present review specifically targeted a clinical psychiatric population [39], and only Luttenberger et al. [47] directly assessed mental health outcomes such as psychological distress or mental health. This gap is notable, given the prevalence of psychological distress and anxiety in children and adolescents [16, 76, 77]. Conditions such as ADHD or ASD, where anecdotal evidence and non-controlled studies suggest climbing’s benefits [70, 72], remain underexplored in controlled trials.

Climbing’s group-based implementation offers unique opportunities for social interaction, trust-building, and cooperation [73]. The studies included in this review examined various aspects of social dynamics, such as alienation [45], social cohesion [47], and barriers self-efficacy in belaying [46]. The concept of alienation is defined by Bronfenbrenner [78] as a lack of a sense of belonging. Alienation can be mitigated through structured climbing programs that provide clear roles and responsibilities, fostering a sense of inclusion. Belaying, a core component of rope climbing, exemplifies social interactions and can enhance trust, collaboration, and communication between climbers and belayers. Krüger and Seng [46] showed a significant increase in barriers self-efficacy in belaying. While in this study this was primarily measured as an individual’s confidence in overcoming challenges, it also inherently reflects trust, collaboration, and communication between the climber and belayer. This duality suggests that belaying extends beyond individual performance, contributing to the development of social skills and interpersonal relationships. A recent qualitative study in an inpatient mental health rehabilitation facility also showed that adolescents of the climbing therapy emphasized the opportunity to foster trust, cooperation and social competence [73]. Similar processes were described in previous literature [79–83]. Especially for individuals facing social or emotional challenges in everyday life, such as those persons with special needs, these group-based interventions may provide a supportive environment to practice and enhance social skills. However, the extent of these benefits likely depends on group size, facilitator support, and participant dynamics.

The studies included in this review varied in group sizes and participant-to-therapist ratios, ranging from 1:1 or 1:2 in children with special needs [39, 48] to larger groups of up to 12 participants [47]. Smaller groups with higher support ratios allowed for individualized guidance and created a highly supportive environment for participants requiring more assistance. In contrast, larger groups, often involving peer-to-peer belaying, relied on peer support and basic instructions [46] fostering social interactions, trust-building, and cooperative learning. However, larger group settings may limit individualized attention, which could disadvantage participants needing more guidance. Further, Gassner et al. [34] noted that social outcomes were often dependent on the length and format of interventions. Long-term programs, such as a one-year-long intervention [31] were more likely to yield significant improvements compared to shorter sessions [34]. Future research should explore how group size and support ratios affect outcomes, particularly in clinical contexts where access to trained professionals is limited. Balancing individualized support with group-based dynamics is critical for optimizing therapeutic climbing programs [80].

The roles of professionals in climbing interventions varied widely, with different qualifications and experience which might also affect the quality of interventions. A study of teachers’ attitudes towards integrating climbing into PE lessons highlighted gaps in their knowledge about who is qualified to teach climbing [84]. These gaps are likely to extend to other professions, underscoring the importance of standardized training to ensure consistent, safe, and effective delivery of climbing (therapy) across diverse settings.

The results of this review highlight the potential of climbing to improve self-efficacy, with two studies showing significant effects on self-efficacy outcomes [46, 48]. Climbing’s impact on self-efficacy has also been studied in adults, showing significant benefits for individuals with mental health challenges like depression. One RCT found therapeutic climbing improved self-efficacy more than home-based exercise and was as effective as cognitive-behavioral therapy [85]. Another study reported acute self-efficacy increases after climbing compared to Nordic walking and sedentary controls [86]. Self-efficacy, crucial for mental health and resilience, is the belief in one’s ability to overcome challenges [58]. Krüger and Seng [46] found self-efficacy improvements primarily in belaying, highlighting trust and collaboration. The short intervention may have limited gains in climbing-specific self-efficacy, as self-efficacy growth depends on consistent mastery [58]. Longer interventions could enhance both specific and generalized self-efficacy. The process-oriented nature of self-efficacy suggests benefits may extend beyond climbing [46], so that repeated successes in climbing could generalize to other life domains, enhancing resilience and coping mechanisms. Nevertheless, as Bandura [58] notes, such generalization is not automatic and depends on the consistency of positive experiences.

Enhanced self-efficacy through climbing may also have implications for broader PA engagement. Studies suggest that self-efficacy at the time of exercise correlates with PA behavior up to 12 months later [87]. Hence, enjoyment of the activity is an even stronger predictor of sustained PA behavior [87], and climbing, as an intrinsically motivating and engaging form of exercise, combines both elements [29, 88]. This is predominantly relevant given the acute effects observed in one included study [39], where climbing elicited stronger affective responses than other interventions in in-patient youth with mental health disorders. These immediate, positive emotional experiences may be especially critical for this population, as they can enhance therapy adherence and foster long-term behavior change. Participants in the study by Mazzoni et al. [48] expressed the desire in continuing climbing, suggesting that the activity’s inherent appeal may encourage long-term participation. Given that few children and adolescents meet PA recommendations [89], climbing may offer a sustainable engagement strategy.

A significant limitation of the current evidence on therapeutic climbing is the lack of studies incorporating active control interventions. Within this review, only Frühauf et al. [39] directly compared therapeutic climbing to other interventions, specifically swimming and occupational therapy. The remaining studies predominantly utilized wait-list control groups [46–48] or groups that received no intervention [45], which limits the ability to discern whether observed benefits are unique to climbing or could be achieved through other PA or therapeutic interventions. In clinical practice, this question holds particular relevance, as climbing therapy is resource-intensive, requiring specialized equipment, trained professionals, and access to appropriate facilities [84]. Demonstrating clear evidence of its unique or added benefits could help enhance the feasibility of integrating therapeutic climbing into broader therapeutic frameworks [79]. Longitudinal studies and randomized controlled trials comparing climbing to other established therapeutic approaches, such as yoga or swimming, are essential to address these gaps [73]. Such research could help clarify therapeutic climbing’s long-term benefits, identify optimal intervention designs, and determine whether its multidimensional nature - incorporating physical, social, and psychological elements - provides distinct therapeutic advantages compared to other PA [75].

A key limitation in current research is the potential influence of expectations on outcomes. Participants’ beliefs about climbing and their ability to choose the intervention may have amplified perceived improvements in self-efficacy and affective responses [90–92]. For example, in the study by Cross [45], participants voluntarily enrolled in the climbing program, which may have fostered a greater sense of personal control over their participation and increased engagement. Additionally, facilitators’ enthusiasm, positive reinforcement, and communication style can shape therapeutic outcomes, making it difficult to distinguish climbing’s intrinsic effects from expectancy-driven improvements [93–95].

Blinding remains a challenge in active therapies, as climbing interventions rely on interaction between facilitators and participants. This social component may play a significant role, potentially outweighing the physical activity itself [96]. Studies using social interaction control groups, such as Bichler et al. [96] on climbing for anxiety and PTSD, have shown smaller effects than those using wait-list controls, highlighting the need for improved control group designs [97]. Future research should aim to account for these influences while recognizing the integral role of social and psychological factors in therapeutic climbing.

### Limitations

The review adhered to a rigorous methodology in accordance with PRISMA guidelines to ensure the accurate identification and evaluation of relevant literature. Clear research questions and predefined eligibility criteria were established prior to the search, minimizing the potential for reviewer bias during the study selection process. This review has several limitations to consider. First, while a thorough search was conducted across various databases, grey literature and unpublished studies were excluded. Restricting the review to published studies was intended to focus on scientifically robust findings, but this may have limited the breadth of the evidence included. Further, the inclusion of both clinical and non-clinical populations may obscure differences in outcomes, particularly within psychological domains. Clinical populations often experience different psychological impacts compared to non-clinical groups, which may influence their responses to interventions like climbing. Therefore, the results should be interpreted with caution, as the effects of climbing might vary based on the population being studied. Lastly, variability in intervention designs and outcomes posed challenges in synthesizing a clear and cohesive picture of the impact of climbing interventions. This heterogeneity, despite efforts to standardize search methods and follow established guidelines, may limit the generalizability of the findings.

## Conclusion

To the best of the authors knowledge this is the first systematic review investigating climbing interventions as an approach to foster mental health in children and adolescents. The results based on five included studies suggest that climbing interventions can have significant positive effects on mental health, and interrelated domains such as emotional regulation, affective states and self-efficacy in children and adolescents. However, the results should be interpreted cautiously due to the limited number of high-quality studies available in this field. Future studies should focus on generating higher-quality evidence to provide more definitive recommendations for practice, incorporating a range of well-designed study types, including RCTs. Additionally, the inclusion of active control groups will be important to allow meaningful comparisons between therapeutic climbing and other PA for greater consistency in population selection, settings, and outcome measures to facilitate clearer and more comprehensive conclusions. Also, the current state of research does not yet provide sufficient evidence to support its routine integration into clinical treatment frameworks. Practical implementation would require more robust evidence to determine its specific therapeutic advantages over other approaches.

## Supplementary Information

Supplementary Material 1.

Supplementary Material 2.

## Data Availability

The data supporting this systematic review are derived from publicly available studies, which are cited in the reference list of this manuscript. No additional datasets were generated or analysed during the study.
