# Supplementary material for: The role of therapeutic climbing in child and adolescent mental health: a systematic literature review
Source: BMC Sports Sci Med Rehabil. 2025 Nov 24;17:376. doi: 10.1186/s13102-025-01417-7 (PMC12750606; doi:10.1186/s13102-025-01417-7)
Supplement: Supplementary file 1 — Supplementary Material 1. [file 13102_2025_1417_MOESM1_ESM.docx]

*Additional file 1: Search Strategy*

**Databases Searched:**

- PubMed
- PsycINFO
- PsycARTICLES
- PubPsych
- Medline
- Web of Science
- CINAHL

**Date of Search:**
3rd December 2024

**Search Period:**
All records available up to 3rd December 2024 were searched without any restrictions on publication date.

**Publication Type:**
Only peer-reviewed articles were included in the search. Dissertations, theses, unpublished literature, and grey literature were excluded.

**Search Terms and Boolean Operators:**
The search combined three main elements: population (children and adolescents), intervention (climbing), and outcomes (mental health and related psychological parameters). Keywords and phrases were adapted per database but followed the structure below. An example search string used (adapted for databases supporting TITLE-ABS-KEY searches) was:

(TITLE-ABS-KEY (Climb* OR boulder* OR rock-climb* OR sport-climb))

AND (Intervention OR therap* OR treatment OR program OR exercise OR sport)

AND (Child* OR adolescent* OR youth OR teenager OR school-aged OR pediatric OR "young people")

AND (Mental health OR mental well-being OR psychological well-being OR emotion* OR stress OR anxiety OR depression OR self-esteem OR self-efficacy OR resilience OR affect* OR mood OR behavior OR quality of life OR coping OR self-harm OR recovery OR ADHD OR "attention-deficit-hyperactivity disorder")

**Language Restriction:**
Only articles published in English or German were considered.
